# Super enhancer associated *RAI14* is a new potential biomarker in lung adenocarcinoma

**DOI:** 10.18632/oncotarget.22165

**Published:** 2017-10-27

**Authors:** Chongze Yuan, Hong Hu, Muyu Kuang, Zongwei Chen, Xiaoting Tao, Shengjian Fang, Yihua Sun, Yawei Zhang, Haiquan Chen

**Affiliations:** ^1^ Department of Thoracic Surgery, Fudan University Shanghai Cancer Center, Shanghai, China; ^2^ Department of Oncology, Shanghai Medical College, Fudan University, Shanghai, China; ^3^ Institutes of Biomedical Sciences, Fudan University, Shanghai, China; ^4^ Department of Thoracic Surgery, Zhongshan Hospital, Shanghai, China

**Keywords:** lung cancer, ChIP-seq, super enhancer, RAI14, targeted therapy

## Abstract

**Purpose:**

Tyrosine kinase inhibitors (TKIs) are widely used to treat lung adenocarcinoma patients with *EGFR* mutations or *ALK*-fusions. However, patients with wild-type genes or TKIs-resistant mutations lack effective therapeutic targets. Extensive studies reveal that super enhancer (SE), a large *cis*-regulatory element, is associated with key oncogenes in a variety of cancers. By comparing the effect of SE on lung adenocarcinoma cell lines with normal cell line, this work attempts to find new biomarkers and potential therapeutic targets for lung adenocarcinoma.

**Experimental Design:**

Chromatin Immunoprecipitation (ChIP) followed by high-throughput DNA sequencing (ChIP-seq) of H3K27ac (acetylation on lysine 27 of histone 3) was performed in lung adenocarcinoma cell lines SPC-A1 and SCH-1153. The differences in SE distribution were then analyzed among SPC-A1, SCH-1153, A549 and normal human lung fibroblasts (NHLF) to identify SE-associated oncogenes. The expression of SE-associated oncogenes was then detected by RNA-seq and further verified in 71 patients by real-time PCR.

**Results:**

SE associated with many new oncogenes in lung adenocarcinoma, among which, *RAI14* was up-regulated in A549 and 31 of 71 patients. High expression of *RAI14* could inhibit cell proliferation, indicating its potential as a new biomarker for lung adenocarcinoma.

## INTRODUCTION

One of the most malignant tumors yet discovered, lung cancer has been the leading cause of mortality of male worldwide. The lung cancer is also the most common cancer and leading cause of death in China, with an incidence of 733.3/100,000 and mortality of 610.2/100,000 separately [1]. Numerous genomic alterations have been found in correlation with oncogenesis and cancer progress, such as *EGFR* mutations, *ALK*-fusions, and *KRAS* mutations. *EGFR* mutations were found in 30% to 60% of Asian patients and 10% to 20% of Caucasian patients with NSCLC (non-small cell lung cancer), while *ALK*-fusions were found in 2% to 5% of patients [2–5]. Patients with *EGFR* mutations or *ALK*-fusions are recommended to receive targeted therapy of tyrosine kinase inhibitors (TKIs), which significantly prevents the progression of cancer and improves patients’ prognosis [6].

*KRAS* mutations maybe the most frequent gene abnormalities in lung adenocarcinoma since about 3% to 8% of Chinese patients and 15% to 25% of Caucasian patients were *KRAS* mutations positive [7–10]. *KRAS* mutations are usually considered as poor prognosis biomarkers mainly because these mutations cause nonresponse to EGFR-TKI treatment [11]. MEK inhibitors have been approved for the treatment of *KRAS* mutations positive colorectal cancer patients, but they did not show priority to docetaxel in patients with previously treated *KRAS* mutations positive NSCLC [12].

In general, the treatment of patients without *EGFR* mutations/*ALK*-fusions or with *KRAS* mutations is still challenging due to the lack of effective therapeutic targets. Super enhancer (SE) is a large *cis*-regulatory element recently put forward which is related to key cell identity genes and diseases. Cancer cell could acquire and activate carcinogenic SE by chromosomal rearrangement, focal amplification and over-expression of transcription factors [13]. Over-expression of SE-associated oncogenes play important roles in tumor pathogenesis. Associated with new oncogenes, abnormal SEs help us find potential therapeutic targets. In this study, we selected 3 lung adenocarcinoma cell lines SPC-A1, SCH-1153 and A549, which were all inappropriate for TKIs due to the lack of *EGFR* mutations/*ALK*-fusions or possessing *KRAS* mutations. With the use of the SE maker H3K27ac (acetylation on lysine 27 of histone 3), we performed Chromatin Immunoprecipitation followed by high-throughput DNA sequencing (ChIP-seq) in SPC-A1 and SCH-1153 [14, 15]. After comparing the ChIP-seq data with online data of A549 and NHLF, we found 1453 tumor-associated SEs. Based on the mRNA expression detected in SE-associated genes in both cell lines and tissue samples, we identified *RAI14* as a novel SE-associated biomarker and potential therapeutic target in lung adenocarcinoma.

## RESULTS

### Super enhancer landscape in SPC-A1, SCH-1153, A549 and NHLF

**Figure 1:**
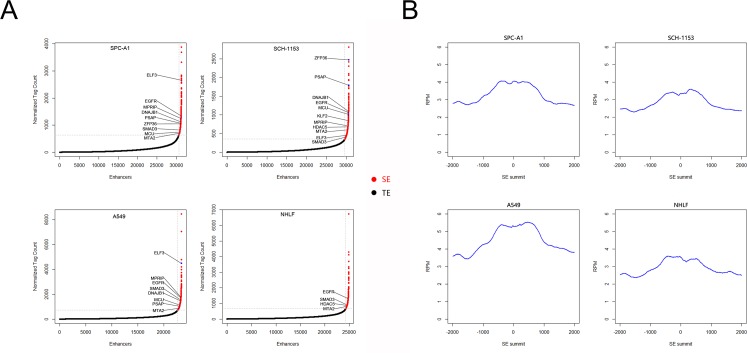
SE scatter plots and histograms of H3K27ac signal distribution in 4 cell lines **(A)**, Scatter plots of SEs in 4 cell lines. All stitched regions were ranked by H3K27ac signal, SE and TE were in different colors as indicated. **(B),** Bimodal H3K27ac signal distribution at identified SE regions. SE: super enhancer, TE: typical enhancer.

**Figure 2:**
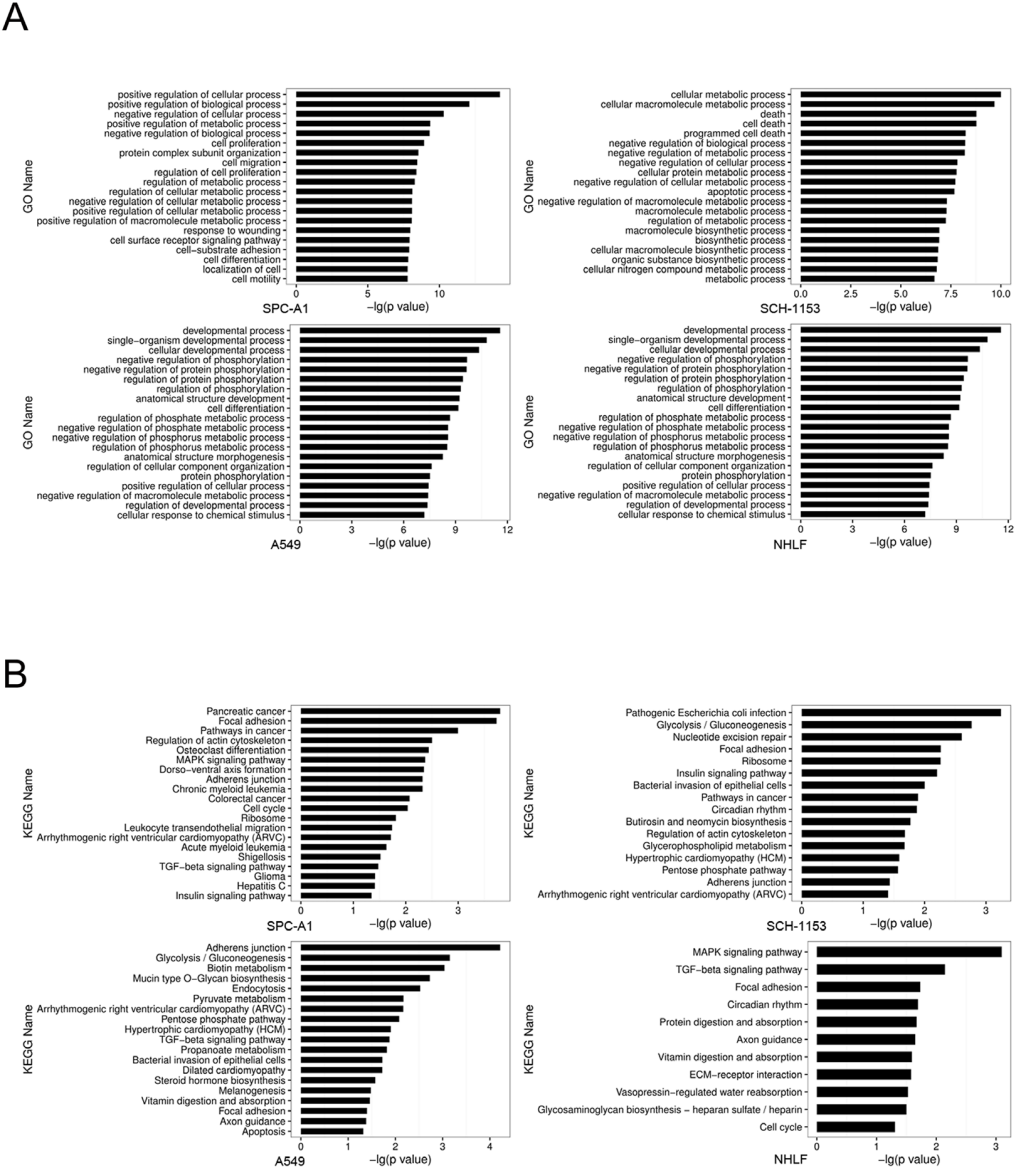
GO and KEGG analysis of SE-associated genes in 4 cell lines **(A),** GO analysis for SE-associated genes in 4 cell lines. Ordinate comments indicated the biological processes that genes may be involved in, the abscissa values mean the statistical significance of processes. All processes listed were statistically significant with p<0.05. **(B),** KEGG analysis for SE-associated genes in 4 cells. Ordinate comments indicated the pathways that genes may be involved in, the abscissa values mean the statistical significance. All the pathways listed were statistically significant with p<0.05.

To investigate the SE distribution in lung adenocarcinoma cells and normal cells, we utilized H3K27ac as our SE marker and performed ChIP-seq in SPC-A1 and SCH-1153 with the use of H3K27ac specific antibody [16–18]. The raw ChIP-seq data of A549 and NHLF using the same of antibody came from ENCODE. The ChIP-seq data of 4 cell lines were then analyzed to identify H3K27ac enrichment regions (peaks). After stitching peaks in 12.5kb and ranking, we identified 655, 984, 701 and 603 super enhancers in SPC-A1, SCH-1153, A549 and NHLF respectively [19, 20] (Figure 1A, 1B). Consistent with previous studies, genes with critical functions were SE-associated in 4 cell lines, such as *ZFP36*, *ELF3*, *SMAD3* and *HDAC5* (Supplementary-1). Furthermore, lots of well-studied oncogenes were also dominated by SE, such as *MTA2*, *EGFR* and *KLF2* (Supplementary-1). In order to understand the biological features of SE, we performed GO and KEGG analysis of SE-associated genes. In GO analysis, compared with NHLF, more tumor-related biological processes were enriched in 3 lung adenocarcinoma cell lines, including cell proliferation, migration and differentiation (Figure 2A). The majority of SE-associated genes enriched in NHLF were essential genes for cell metabolism and development, which were also enriched in SPC-A1, SCH-1153 and A549. Similar to GO analysis, critical pathways like cell cycle and focal adhesion were found in 4 cell lines, while more tumor-related pathways were enriched in SPC-A1, SCH-1153 and A549 (Figure 2B).

### Tumor-associated SEs control oncogenes’ expression in lung adenocarcinoma

**Figure 3:**
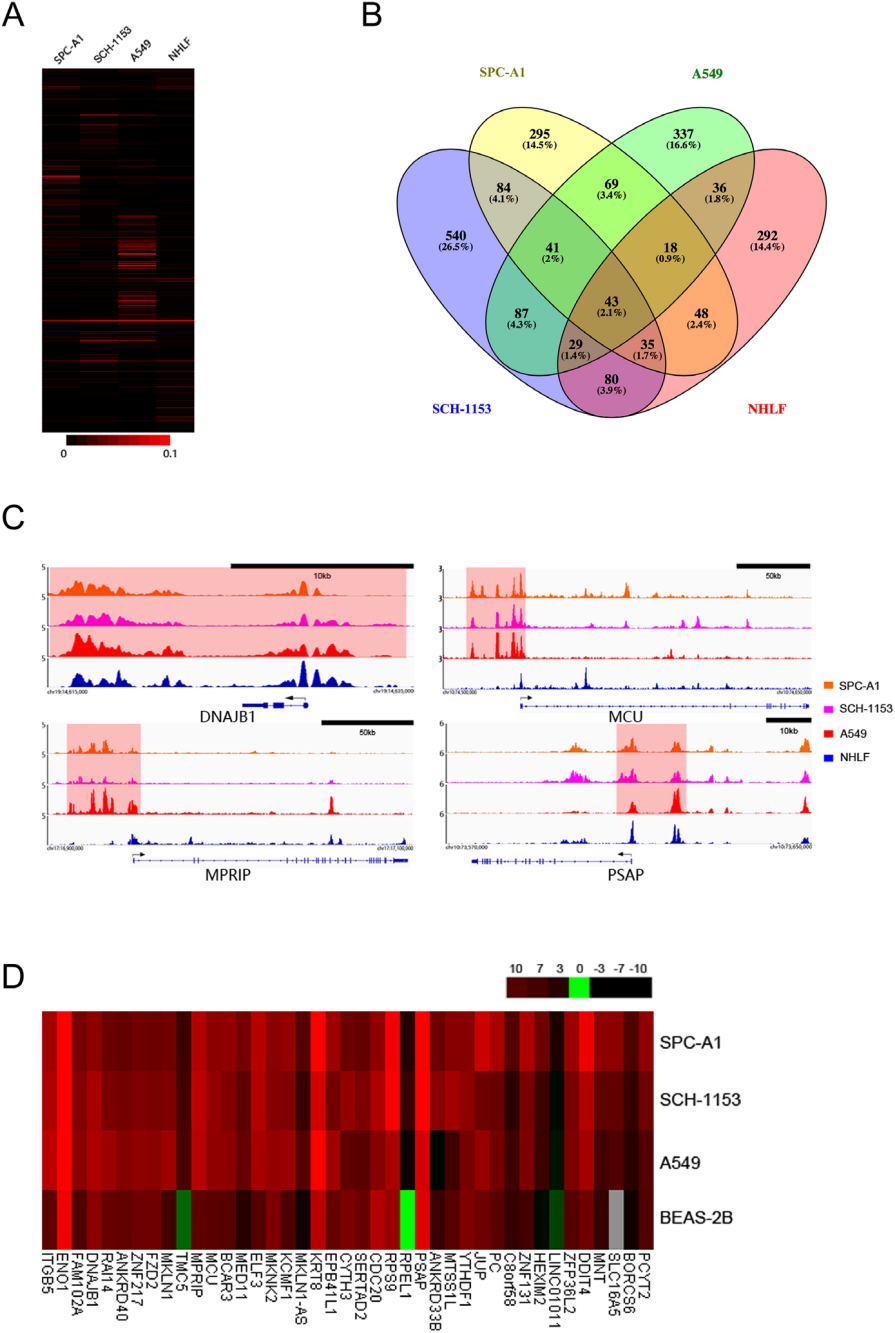
The H3K27ac enrichment differences, SE distribution, ChIP-seq binding profiles and associated genes’ expression in 4 cell lines **(A),** Heat map showed H3K27ac enrichment at all identified SE regions in 4 cell lines. **(B),** Venn diagram showed SE distributions in 4 cell lines. The number and percentage of SEs shared by different cell lines were as indicated. **(C),** ChIP-seq binding profiles of H3K27ac at *DNAJB1*, *MCU*, *MPRIP* and *PSAP* in 4 cell lines as indicated. Gene models were depicted below the binding profiles and SEs were marked out with light red background. **(D),** Heat map showed expression of 38 tumor-specific SE-associated genes, *RAI14*, *ZNF131* and *TMC5* in SPC-A1, SCH-1153, A549 and BEAS-2B.

SE is a complicated element that is occupied by high density of transcription factors, mediators and other components, which are all necessary to its function of transactivation [19]. SE is not immutable, extracellular or intracellular environmental alterations could change SE landscape and influence gene transcription [21, 22]. To comprehensively investigate SE diversity in 4 cell lines, we performed H3K27ac enrichment analysis at all identified SE regions (Figure 3A, Supplementary-2). As shown by our results, a small portion of identified SE regions exhibited similar H3K27ac enrichment, but at most regions, significant enrichment diversities were observed across 4 cell lines. In our study, we identified 2034 SE regions in 4 cell lines, among which 1464 SEs were cell-type specific (Figure 3B). These cell-type specific SEs were associated with various genes and may collaboratively contribute to cell features. 43 of 2034 SEs, only 2.1% of all identified SEs were shared by 4 cell lines, regulating genes which were involved in critical cell functions such as transcriptional regulation, cell proliferation and signal transduction. To search new oncogenes, we mainly focused on the 1453 tumor-associated SEs which were not identified in NHLF, especially the 41 SEs shared by 3 lung adenocarcinoma cell lines. The SE-associated transcriptional dysregulation may facilitate tumor pathogenesis. And to efficiently identify potential SE-associated oncogenes, we hypothetically regarded these 41 SEs as tumor-specific SEs. In 41 tumor-specific SEs, several SEs with significant H3K27ac enrichment were close to previously reported tumor-related genes, like *MCU*, *MPRIP* and *PSAP* (Figure 3C, Supplementary-2).

To investigate whether these potential oncogenes exhibited abnormal expression in lung adenocarcinoma, we performed RNA-seq in SPC-A1, SCH-1153, A549 and human bronchial epithelial cell line (BEAS-2B), instead of NHLF (Supplementary Figure 1). In 38 tumor-specific SE-associated genes, 37 genes showed significantly elevated expression in at least 1 cancer cell line, including *DNAJB1*, *MCU*, *MPRIP* and *PSAP* (Figure 3D). *DNAJB1* is a well-studied oncogene that could promote cell proliferation and has also been reported as an SE-associated oncogene in esophageal squamous cell carcinoma [23]. In our study, *DNAJB1* was also SE-associated, but the H3K27ac enrichment just slightly decreased in NHLF (Supplementary-2). In a variety of cancers, these 4 up-regulated genes (*DNAJB1*, *MCU*, *MPRIP* and *PSAP*) have been reported to be related to drug-resistance processes, cell death, cancer invasion and metastasis [24–27].

Besides these 37 genes, *RAI14* (SE-associated in A549), *ZNF131* (SE-associated in SPC-A1) and *TMC5* (non-SE-associated) were up-regulated in lung cancer, which were also potential biomarkers of lung adenocarcinoma (Figure 3D). The complexity of transcriptional regulation is highly likely to be a main reason causing the deviation between SE prediction and associated gene expression, as our results showed. However, our results still proved the SE's intensive regulation of transcription was irreplaceable.

### *RAI14* is a potential biomarker of lung adenocarcinoma

**Figure 4:**
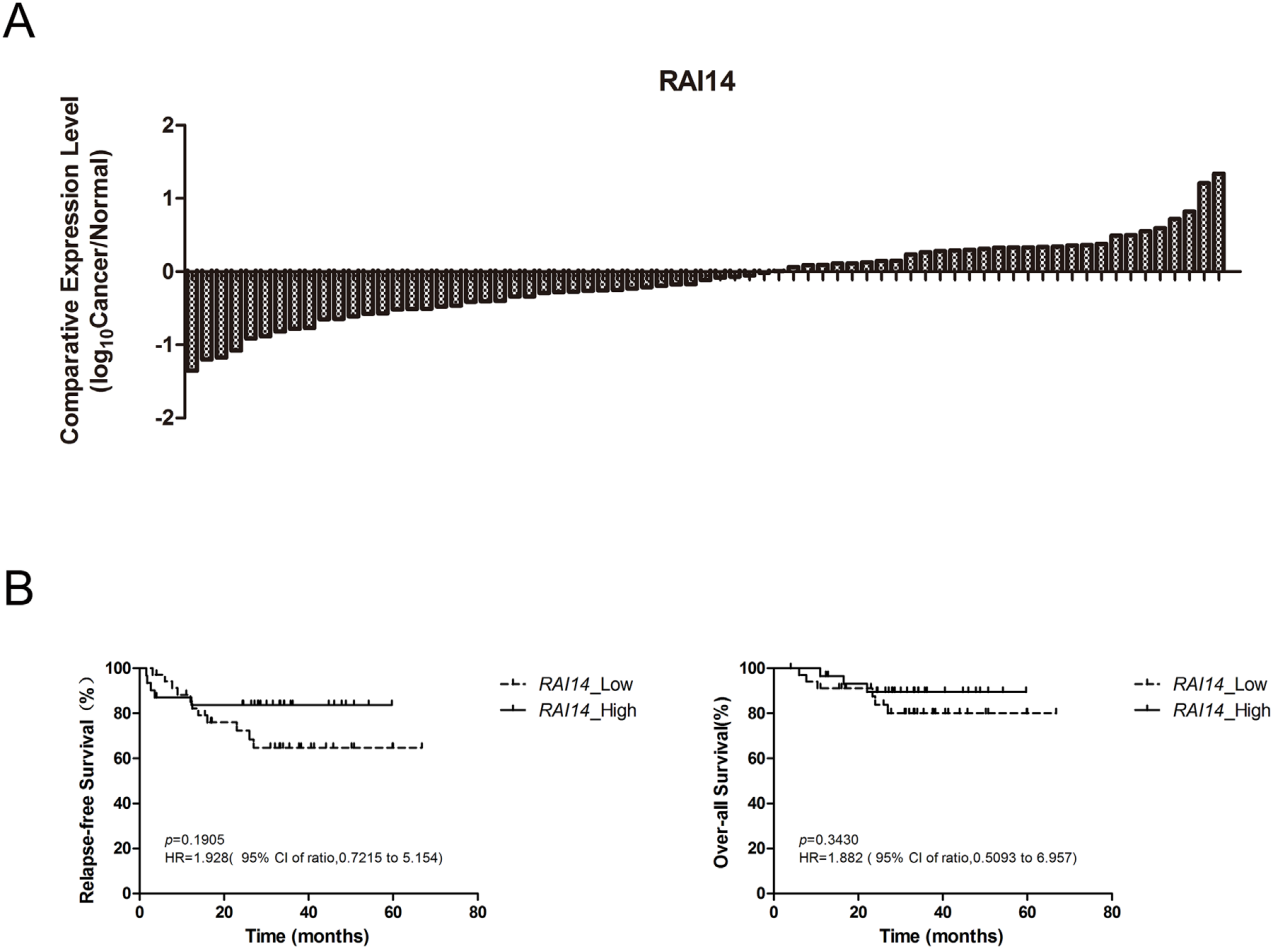
*RAI14* expression status in 71 patients and cumulative probability of relapse-free survival and over-all survival for patients with lung adenocarcinoma **(A),** *RAI14* expression status in 71 patients. The final result was displayed in the form of log_10_ as the Y-Axis label indicated. **(B),** Relapse-free survival and over-all survival for 66 patients with lung adenocarcinoma.

Our results of SE distribution and associated gene expression revealed several oncogenes as potential biomarkers. To evaluate the possibility for clinical application, we tested the expression of *DNAJB1*, *MCU*, *MPRIP*, *PSAP*, *RAI14*, *ZNF131* and *TMC5* in 71 lung adenocarcinoma samples and paired adjacent normal tissue samples (>2 cm away from the tumor) from patients who underwent lobectomy or pneumonectomy. To most genes, gene expression was down-regulated or slightly up-regulated in our patients (Supplementary Figure 2). However, we identified *RAI14* as a new biomarker because *RAI14* was up-regulated in 31 of 71 patients (Figure 4A).

*RAI14* has not been applied as a biomarker or therapeutic target yet, there is even no medicine specifically targeting it. In a study performed by Yi-Chiung Hsu *et al*, although *RAI14* was not studied separately, as 1 of 8 invasion-associated genes, it showed significant gene-drug correlation with paclitaxel, docetaxel and erlotinib and correlation with patients’ relapse-free survival time [28]. In our study, it seemed that *RAI14* expression was higher in elderly patients (>60 years old) and male patients, but the differences were not significant (Table 1). The expression of the other 6 genes we tested was not related to patients’ age or gender either (Supplementary-3). The effect of *RAI14* on patients’ prognosis (relapse-free survival, RFS and over-all survival, OS) was also analyzed. It seemed that *RAI14* expression up-regulated patients (*RAI14*_High) got better prognosis, however, the differences were not significant, neither on PFS nor on OS (Figure 4B).

**Table 1:** Patient characteristics and *RAI14* expression status

Characteristics	No. of patients	Mean expression level
		*RAI14*
**Age**		
** ≤60**	28	1.18
** >60**	41	2.12
	P value	0.24
**Gender**		
** Male**	22	1.97
** Female**	47	1.63
	P value	0.68

### SE-induced *RAI14* high expression inhibits cell proliferation

**Figure 5:**
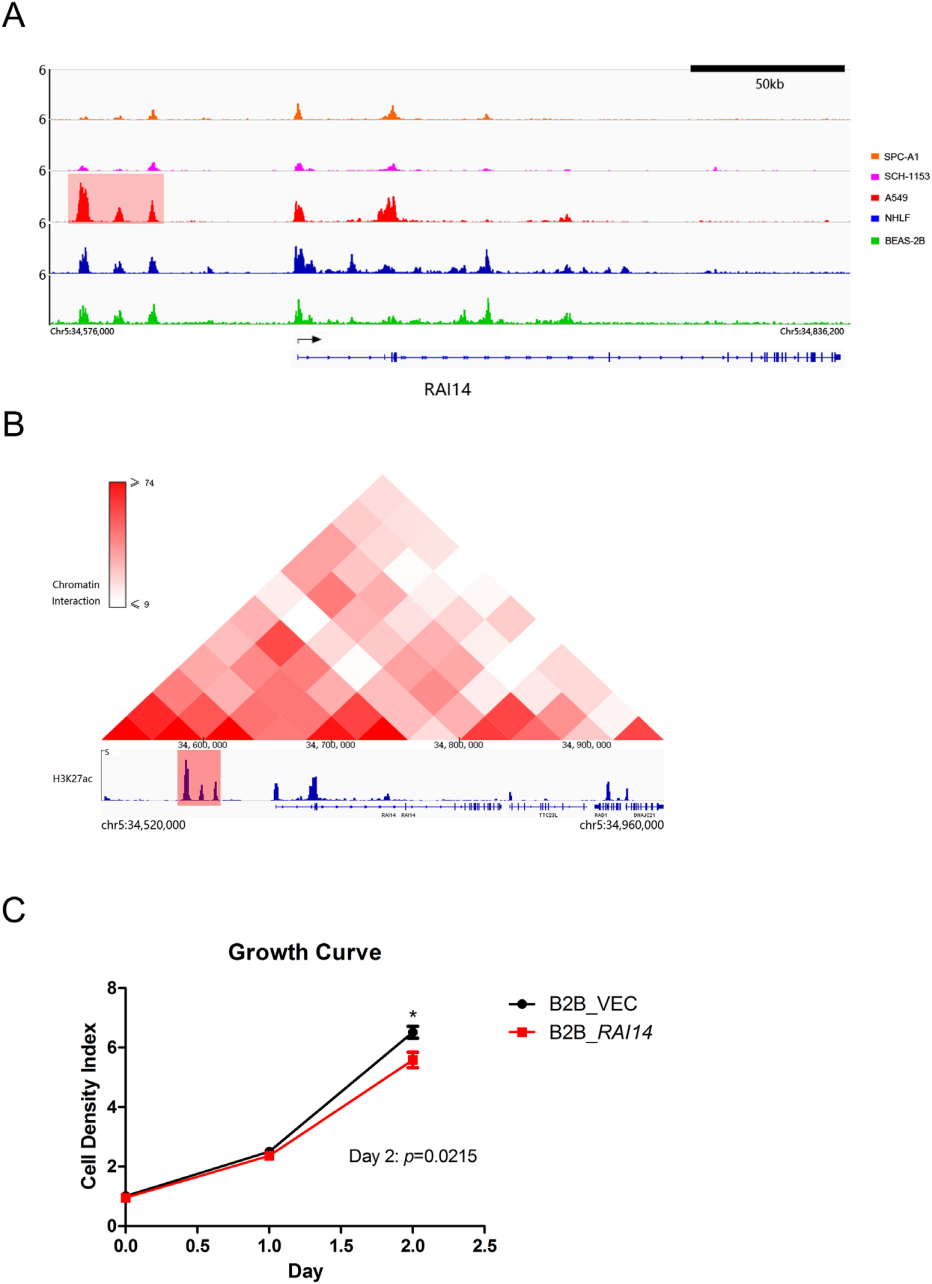
ChIP-seq binding profiles, Hi-C heat map at *RAI14* in A549 and growth curve **(A),** ChIP-seq binding profiles of H3K27ac at *RAI14* in 5 cell lines as indicated. Gene model was depicted below the binding profiles. The SE was marked out with light red background. **(B),** Hi-C heat map and ChIP-seq binding profile of H3K27ac at *RAI14* in A549. The color bar indicated the interaction frequency between two regions of chromatin. The SE associated with *RAI14* is marked out with light red background. **(C),** Growth curve of *RAI14* over-expression BEAS-2B cell and normal BEAS-2B cell.

As we performed RNA-seq in BEAS-2B instead of NHLF, to get a more reliable conclusion, we then identified SEs in BEAS-2B. Our ChIP-seq results indicated a SE located at 45kb upstream of *RAI14* in A549 but not in SPC-A1, SCH-1153, NHLF or BEAS-2B and the SE could give rise to extremely high expression of *RAI14* (Figure 5A, Figure 3D). To prove the hypothesis of SE-induced *RAI14* high expression true, we analyzed the Hi-C data and ChIP-seq data of A549 from ENCODE and found frequent interactions between *RAI14* gene body and the upstream SE region, which indicated the up-regulated gene expression was caused by this upstream SE (Figure 5B).

To understand the biological function of *RAI14*, we infected BEAS-2B cells by lentivirus to stably express *RAI14*. *RAI14*, also known as *NORPEG*, has been reported to promote cell proliferation and shorten ovarian cancer cell cycle as a downstream gene of *NR2F2* [29, 30]. However, in our study, over-expression of *RAI14* could inhibit proliferation of BEAS-2B cells, which may result in better prognosis of *RAI14* expression up-regulated patients (Figure 5C).

## DISCUSSION

Precise diagnosis and targeted therapy are more and more attractive for lung cancer treatment. Even more and more genomic alterations were found, it's still hard to find new biomarker genes because most of these abnormalities are irrelevant with tumorigenesis. The discovery that SE in cancers usually associates with key oncogenes provides us a feasible method to look for new potential oncogenes [19]. Cancer cell could gain carcinogenic SEs through a variety of mechanisms during cancer pathogenesis [13]. Moreover, SEs in cancers are more sensitive to perturbation than typical enhancers (TEs), so the expression of associated genes is impaired more significantly than other genes [31–35]. Previous studies of SE render the strategies to inhibit the expression of SE-associated oncogenes by damaging their SEs.

ENCODE and Roadmap Epigenomics Project have gathered H3K27ac ChIP-seq data of different cells and tissues, but SPC-A1 and SCH-1153 in our study are investigated for the first time [36–38]. In current studies, only a few known driver mutations are proved to be related to SE alterations. Oncogenic SEs mainly generate from gene fusions, non-coding region mutations, focal amplifications and deletions [39–41].

The deviation between cell line and tissue is a challenge in our study and also a problem to other researchers. In our study, even though most part of oncogenes showed significantly increased expression in cell, the expression status couldn't be verified in tissue. And *RAI14* was up-regulated in 31 of 71 patients, but it was only associated with SE in A549. As the deviation is hard to eliminate, direct SE researches in tumor samples are necessary for more valuable discoveries and further clinical applications. Based on the comprehensive study of SE distribution and patients’ characteristics, we can create new methods for tumor classification and prognosis prediction. For example, Lin *et al* have successfully found subgroup-specific SEs and revealed cellular origins by analyzing SE-regulated TFs differences in medulloblastoma [42].

For the first time, we described the SE landscape in SPC-A1 and SCH-1153 and identified new SE-associated oncogenes. After verifying the expression of potential oncogenes in both cell lines and tissue samples, we finally found the potential of *RAI14* as a new SE-associated oncogene and a biomarker for patients who were not suitable for TKIs treatment. Our method of oncogene discovery may be applied to study other cancers.

## MATERIALS AND METHODS

### Cell lines and cell culture

SPC-A1 is a commercial lung adenocarcinoma cell line derived from a Chinese patient without *EGFR* mutations, *ALK*-fusions or *KRAS* mutations. SCH-1153 is a primary lung adenocarcinoma cell line derived from a *KRAS* mutation (G12C) positive Chinese patient in our department, which has been stably cultured for more than 50 generations. A549 is a commercial lung adenocarcinoma cell line derived from a *KRAS* mutation (G12S) positive Caucasian patient. BEAS-2B is a commercial human bronchial epithelial cell line we used to instead NHLF, since NHLF is primary normal human lung fibroblasts which is hard for us to acquire.

SPC-A1, SCH-1153 and A549 cells were cultured in RPMI-1640 medium (Corning, 10-040-CVR) suppled with 10% fetal bovine serum (Gemini, 900-108), penicillin (100U/ml, gbico, 10378-016), streptomycin (100mg/ml, gbico, 10378-016) and L-Glutamine (2mM, gbico, 10378-016) at 37°C with 5% CO_2_. BEAS-2B cells were cultured in DMEM medium (Corning, 10-013-CVR) suppled with 10% fetal bovine serum (Gemini, 900-108), penicillin (100U/ml, gbico, 10378-016), streptomycin (100mg/ml, gbico, 10378-016) and L-Glutamine (2mM, gbico, Corning, 10378-016) at 37°C with 5% CO_2_.

BEAS-2B cells were infected by *RAI14*-over-expression lentivirus for 48 hours and then cultured in DMEM medium with 1ug/ml puromycin for 48 hours.

### ChIP-seq

SPC-A1, SCH-1153 and BEAS-2B cells were collected when the confluence was about 90%. Every 20-50 million cells were crosslinked in 20-50ml culture medium with 1% formaldehyde for 10 minutes at room temperature. The cell pellet was collected and washed with cold PBS, snap frozen in liquid nitrogen and stored at −80°C or processed to next step. The cell pellet was dissolved in lysis buffer on ice for 30 minutes and sonicated with Bioruptor UCD-200 (Diagenode, Brussels). The supernatant was collected and mixed with 30μl H3K27ac antibody (Abcam, ab4729) coupled protein A dynabeads (Novex, 10002D) for 6 hours at 4°C. The dynabeads were collected and washed as the previous method described [43]. The chromatin was eluted at 65°C and then reverse-crosslinked at 65°C over-night. The DNA was purified as user manual (Tiangen, DP214-03) and processed to library preparation for next-generation sequencing as instruction manual (NEBNext^®^ Ultra™ II DNA Library Prep Kit for Illumina^®^, E7645S). The sequencing was performed on HiSeq X Ten System (Illumina, Inc).

### ChIP-seq data analysis

ChIP-seq data of A549 and NHLF was obtained from ENCODE (wgEncodeEH003118 and wgEncodeEH000097). ChIP-seq reads were aligned to human reference genome (build GRCh37/hg19) using BWA and peak calling was accomplished by MACS2 (Model-Based Analysis of ChIP-seq) to identify regions of H3K27ac enrichment over background (input) (Supplementary-6) [44]. We took a p-value threshold of 10^−9^ in data analysis. We used IGV to visualize the bigwig files [45, 46]. We used HOMER to identify super enhancers, which emulated the original strategy used by the Young lab [19, 20, 47]. The peaks in 12.5kb were stitched together into larger regions and the signal score was measured by their normalized H3K27ac reads and input reads. Then all regions were ranked by their signal score, and the region passed the point where the slope was 1 was classified as SE. All SE regions were annotated to the nearest Ensemble gene using HOMER. H3K27ac enrichment difference was analyzed with GFOLD [48].

### Hi-C data analysis

Hi-C data of A549 was obtained from ENCODE3, generated by Dekker Laboratory (ENCFF478EAB, ENCFF319AST, ENCFF805BPJ and ENCFF582XKW). The heat map was generated by 3D Genome Browser (http://www.3dgenome.org.).

### Tissue samples

We surgically harvested 71 lung adenocarcinoma samples and paired adjacent normal tissue samples (>2 cm away from the tumor) from patients who underwent lobectomy or pneumonectomy from August 2011 to January 2013. None received preoperative treatments. The information of patients was listed in Supplementary-4. The consent form was signed by every patient or his/her legal representative. The study was approved by the Ethics Committee of Shanghai Cancer Hospital of Fudan University. After resection, part of each tissue was immediately frozen in liquid nitrogen for RNA extraction.

### RNA extraction and real-time PCR

Cell and tissue samples were collected and dissolved in TRNzol (Tiangen, DP405) for total RNA extraction as user manual. For each sample, 2μg total RNA was reversely transcribed using FastQuant RT Kit (Tiangen, KR106). The primer sequences for real-time PCR were listed in Supplementary-5. SE-associated genes and *GAPDH* were amplified for 40 cycles on QuantStudio™ 6 Flex (Applied Biosystems, USA) using SuperReal PreMix Plus (SYBR Green, Tiangen, FP205). The following program: 95°C for 15 minutes, 95°C for 10s, 60°C for 32s was applied for amplification. Quantification was calculated using comparative Ct method. Gene expression was firstly normalized by the *GAPDH* expression in each tissue sample and expression status of each patient was calculated by the following method: (normalized expression in the tumor)/ (normalized expression in normal tissue).

### RNA-seq and RNA-seq data analysis

RNA-seq libraries were prepared as instruction manual (NEBNext^®^ Ultra^™^ RNA Library Prep Kit for Illumina^®^, E7530S). The sequencing was performed on HiSeq X Ten System (Illumina, Inc). RNA-seq reads were aligned to human reference genome (build GRCh37/hg19) using STAR [49]. Expression levels of genes and transcripts were compared using Cufflinks [50].

### Growth curve

CCK8 kit (Yise, CK800-100) was used to make growth curves as user manual. Cells were cultured in 96 well plate and cell density was detected at day 0, day 1 and day 2 for each well.

### Statistical analysis

Statistical comparisons of groups defined by patients’ clinical features with respect to the expression levels of the genes were based on the Unpaired T-test for two groups. All analysis were performed by GraphPad Prism 5 (GraphPad Software, Inc, CA 92037 USA). Statistical comparisons of survival curves were based on Gehan-Breslow-Wilcoxon Test. Statistical comparisons of cell density were based on the Unpaired T-test for two groups. For all the tests, 3 significance levels (^*^, P<0.05; ^**^, P<0.01; ^***^, P<0.001) were applied.

## SUPPLEMENTARY FIGURES AND TABLES











## Abbreviations

SE: super enhancer
TE: typical enhancer
TKIs: tyrosine kinase inhibitors
ChIP-seq: Chromatin Immunoprecipitation followed by high-throughput DNA sequencing
NSCLC: non-small cell lung cancer
