# Supplementary material for: Super enhancer associated RAI14 is a new potential biomarker in lung adenocarcinoma
Source: Oncotarget. 2017 Oct 27;8(62):105251–61. doi: 10.18632/oncotarget.22165 (PMC5739635; doi:10.18632/oncotarget.22165)
Supplement: Supplementary file 1 [file oncotarget-08-105251-s001.pdf]

Super enhancer associated *RAI14* is a new potential biomarker in lung adenocarcinoma

SUPPLEMENTARY MATERIALS

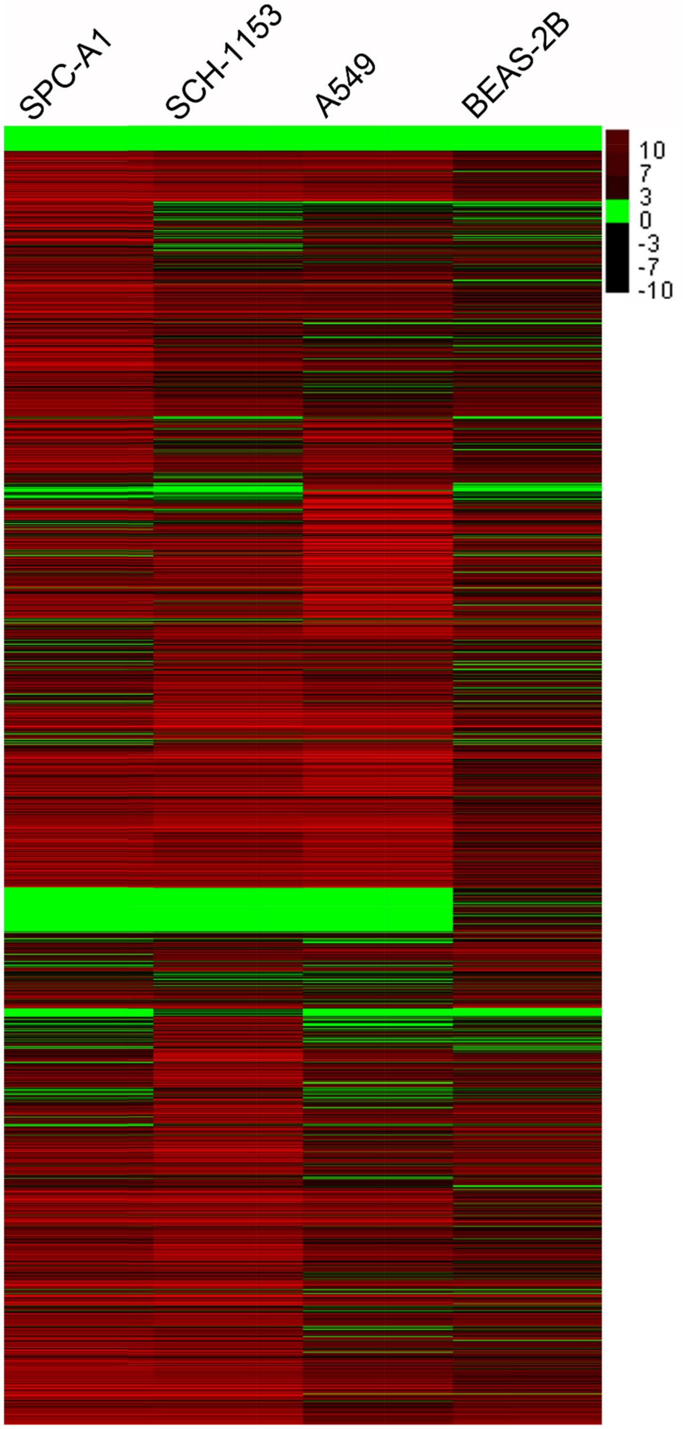

Supplementary Figure 1: Heat map showed expression of SE-associated genes in 4 cell lines.

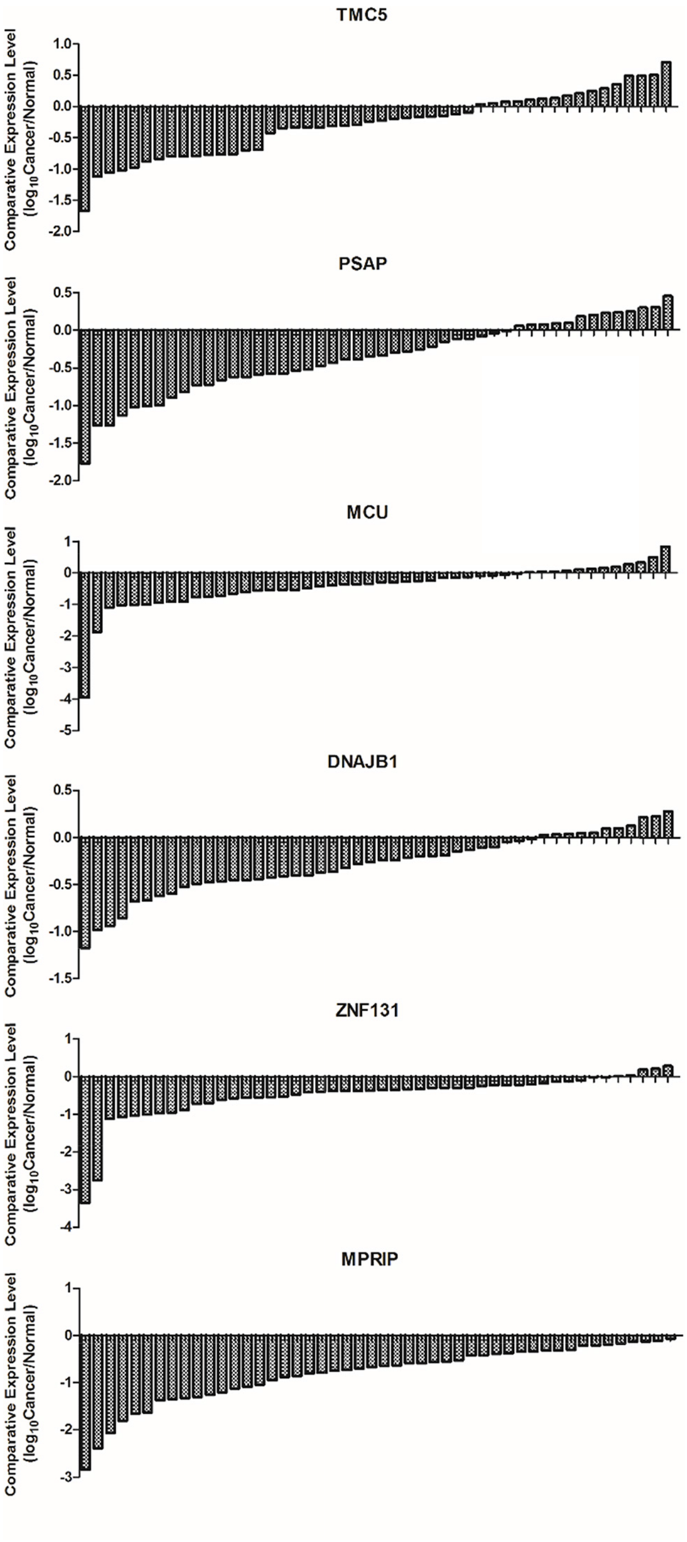

Supplementary Figure 2: *TMC5, PSAP, ZNF131, MCU, DNAJB1, ZNF131, MPRIP* expression status in 48 lung adenocarcinoma samples and paired adjacent normal tissue samples. The expression in lung adenocarcinoma was compared with expression in paired adjacent normal tissue. The final results were displayed in the form of log_{10} as the Y-Axis label indicated.

Supplementary Table 1: Super enhancer associated genes in cell lines

See Supplementary File 1

Supplementary Figure 2: H3K27ac enrichment differences in 4 cell lines

See Supplementary File 2

Supplementary Table 3: Patient characteristics and gene expression status. Gene expression level was not correlated with patients' age or gender (P>0.05)

Characteristics	No. of patients	Mean expression level					
		DNAJB1	*MCU*	*MPRIP*	*PSAP*	*ZNF131*	*TMC5*
**Age**							
**≤60**	21	0.710	0.618	0.260	0.556	0.514	0.835
**>60**	27	0.618	0.906	0.292	0.819	0.495	0.976
	P value	0.483	0.367	0.653	0.168	0.876	0.640
**Gender**							
**Male**	10	0.578	1.17	0.238	0.569	0.529	1.05
**Female**	38	0.679	0.676	0.289	0.740	0.496	0.879
	P value	0.525	0.200	0.554	0.466	0.823	0.644

Supplementary Table 4: Patients clinical information

See Supplementary File 3

Supplementary Table 5: Primer sequences

See Supplementary File 4

Supplementary Table 6: ChIP-seq Reads Mapping Summary

Reads Mapping	SCH-1153_H3k27ac	SCH-1153_input
Original total reads	129.0M	150.5M
Unique mapped reads	116.8M(90.52%)	130.4M(86.66%)
**Reads Mapping**	**SPC-A1_H3k27ac**	**SPC-A1_input**
Original total reads	138.5M	169.9M
Unique mapped reads	125.5M(90.39%)	145.4M(85.59%)
**Reads Mapping**	**A549_H3k27ac**	**A549_input**
Original total reads	59.6M	188.9M
Unique mapped reads	50.1M(84.04%)	150.6M(79.71)
**Reads Mapping**	**NHLF_H3k27ac**	**NHLF_input**
Original total reads	22.4M	20.6M
Unique mapped reads	16.4M(73.3%)	12.5M(60.94%)
**Reads Mapping**	**BEAS-2B_H3k27ac**	**BEAS-2B _input**
Original total reads	173.2	88.9
Unique mapped reads	89.8(51.8%)	40.7M(45.72%)
