# Supplementary figures and images for: Super enhancer associated RAI14 is a new potential biomarker in lung adenocarcinoma
Source: Oncotarget. 2017 Oct 27;8(62):105251–61. doi: 10.18632/oncotarget.22165 (PMC5739635; doi:10.18632/oncotarget.22165)

**Supplementary 3.**

Heat map showed expression of SE-associated genes in 4 cell lines.


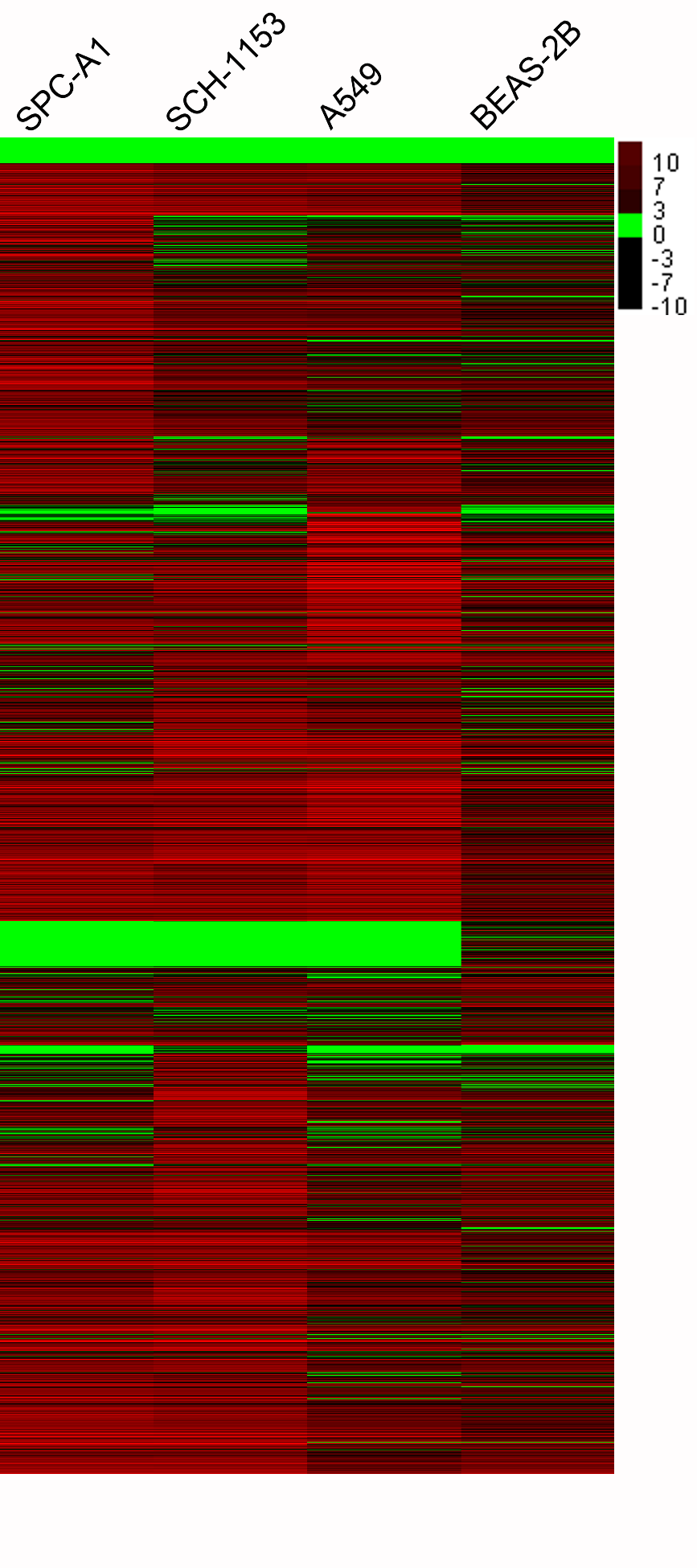

Supplement: Supplementary file 4 [file oncotarget-08-105251-s004.docx]
